# Complete Tooth Loss and Allostatic Load Changes Later in Life: A 12-Year Follow-Up Analysis of the English Longitudinal Study of Ageing

**DOI:** 10.1097/PSY.0000000000000925

**Published:** 2021-04-01

**Authors:** Cesar de Oliveira, Wael Sabbah, Ione Jayce Ceola Schneider, Eduardo Bernabé

**Affiliations:** From the English Longitudinal Study of Ageing (ELSA), Department of Epidemiology and Public Health (Oliveira), University College London; Division of Population and Patient Health, Dental Institute (Sabbah, Bernabé), King’s College London, London, United Kingdom; and Health Science Department and Rehabilitation Post-graduate Program (Schneider), Federal University of Santa Catarina, Araranguá, Santa Catarina, Brazil.

**Keywords:** tooth loss, allostatic load, biomarkers, metabolism, fruit and vegetable consumption, aging, **AL** = allostatic load, **CI** = confidence interval, **ELSA** = English Longitudinal Study of Ageing, **LME** = linear mixed effects, **SD** = standard deviation

## Abstract

**Objective:**

There is little evidence of the association between complete tooth loss and allostatic load (AL). We investigated, firstly, the association between complete tooth loss and changes in AL for 12 years among older English adults. A second aim was to explore the role of fruit and vegetable consumption in explaining the aforementioned association.

**Methods:**

AL was calculated for 2430 English Longitudinal Study of Ageing cohort (2004/5–2016/17) participants 50 years and older based on nine biomarkers: systolic and diastolic blood pressures, glycated hemoglobin, high- and low-density lipoprotein cholesterol, triglycerides, fibrinogen, C-reactive protein, and waist circumference. The exposure was complete tooth loss. Participants were classified as dentate or edentulous. A linear mixed-effects model was fitted to model the 12-year change in AL score and its association with complete tooth loss after adjustments for confounders (demographic factors, socioeconomic position, and health behaviors).

**Results:**

Around 11% of the participants were edentulous. Complete tooth loss was positively associated with baseline AL scores but not with its rate of change over time. The predicted mean AL scores were 3.60 (95% confidence interval [CI] = 3.53–3.68) and 3.98 (95% CI = 3.76–4.21) as well as 4·28 (95% CI = 4·18, 4·39) and 4·66 (95% CI = 4·42, 4·90) for dentate and edentulous participants, at baseline and end of follow-up, respectively. Fruit and vegetable consumption was not associated with baseline AL or its rate of change.

**Conclusions:**

Complete tooth loss was associated with baseline AL score but not with its development over time, whereas the consumption of fruit and vegetables did not help to explain this association. Both conditions may share common determinants earlier in life.

## Abstract 2

Supplemental digital content is available in the text.

## INTRODUCTION

Allostatic load (AL) is the physiological wear and tear that the body sustains throughout the life course. Repeated exposures to chronic stressors trigger biological responses to cope with these stressors, leading to wear and tear on the immune, cardiovascular, metabolic, and nervous systems, and this is primarily marked by elevated epinephrine and cortisol levels (1). AL has been found to longitudinally predict self-rated health, physical function, frailty, and, ultimately, mortality (2). It also shows gradients along markers of socioeconomic position (3). AL is therefore useful not only as a risk factor for morbidity, frailty, and mortality but also in understanding the mechanism of aging throughout the life course (4). There is little evidence on the relationship between AL and oral health with most of the research arguing that AL predicts periodontal disease (5).

On the other hand, poor oral health, particularly complete tooth loss, and periodontal diseases are considered an early marker of frailty (6), cognitive decline (7), metabolic syndrome (8–11), and mortality (12). The association between oral health and metabolic syndrome is particularly significant because the markers of AL include almost all markers of metabolic syndrome.

Although earlier studies have depicted socioeconomic adversity and accumulation of health risk behaviors through life-span as risk factors for the accumulation of biological markers of AL (13,14), it is possible that oral health, particularly complete tooth loss, is a potential and often neglected risk factor or marker for the accumulation of AL. Complete tooth loss could impact some of the risk behaviors related to AL such as poor diet (15,16). Diet is an important component of a healthy life because it has a role in the etiology and thus prevention of many chronic conditions such as obesity, cardiovascular disease, diabetes, and cancer among other chronic conditions (17,18). Tooth loss reduces masticatory function and chewing ability, which in turn can limit food choices and variety in the diet (19). For these reasons, dietary intake has been regarded as an intermediate in the pathway between tooth retention and a number of diet-related chronic diseases (20). Complete tooth loss could also be an early marker of AL because it is linked to adverse socioeconomic conditions and risk behaviors (21,22). One of the most consistent dietary correlates of tooth loss is a lower consumption of fruits and vegetables (23), which is also inversely associated with AL scores (13).

Given that complete loss of all teeth is still relatively common in Britain with 15% of adults aged 65 to 74 years, 30% of those aged 75 to 84 years, and 47% of those 85 years and older edentulous (24), we set out to test whether complete tooth loss is associated with changes in AL over a 12-year period among older English adults. A second aim was to explore the role of fruit and vegetable consumption in explaining the aforementioned association.

## METHODS

### Study Population

We used data from the English Longitudinal Study of Ageing (ELSA), a nationally representative panel study of adults aged 50+ years living in private households in England. ELSA started in 2002–2003 (wave 1) with a sample of 11,391 individuals drawn from households that had participated in the Health Survey for England in 1998, 1999 or 2001 (known as ELSA wave 0). After wave 0, there have been eight waves of data collection with follow-up interviews conducted biannually and health examinations conducted every 4 years.

For this study, we pooled together data on complete tooth loss collected in wave 0 with data on AL collected in waves 2 (2004–2005), 4 (2008–2009), 6 (2012–2013), and 8 (2016–2017). There were 2511 participants with complete tooth loss data in wave 0 and AL data for at least two of the aforementioned waves. Of them, 81 were excluded because of missing data on confounders (fruit and vegetable consumption, 64; wealth, 15; smoking, 1; alcohol consumption, 1). Therefore, the analytical sample included 2430 participants (717 had AL data on 4 waves, 793 on 3 waves, and 920 on 2 waves).

All ELSA participants gave written informed consent. The National Research and Ethics Committee granted ethical approval for all the ELSA waves (http://www.nres.npsa.nhs.uk/; MREC/01/2/91). This study followed the Strengthening the Reporting of Observational Studies in Epidemiology reporting guideline.

### Variables

#### Allostatic Load

The outcome was AL, which was measured four times every 4 years. As a measure of multisystem dysregulation, AL should include biomarkers of the different systems that are thought to be affected by chronic stress exposure. AL was calculated based on nine biomarkers: systolic and diastolic blood pressures (cardiovascular system), high- and low-density lipoprotein cholesterol and triglycerides (lipid metabolism); fibrinogen and C-reactive protein (inflammation), glycated hemoglobin (glucose metabolism), and waist circumference (body fat). Each biomarker was dichotomized according to clinical cutoff points (Table S1, Supplemental Digital Content, http://links.lww.com/PSYMED/A724), and a score for each participant was computed based on the number of biomarkers for which they were above the cutoff (risk category). This AL score ranged from 0 to 9, with higher values indicating higher multisystem physiological dysregulation. We accounted for current medication (2,25). Participants with missing values in more than half (≥5) of the 9 biomarkers were treated as missing when calculating the AL score. For those with <5 missing biomarkers, we averaged the available dichotomous indicators and multiplied this average by 9 to produce an equivalent scale range from 0 to 9 (26). The same approach was also used with all participants in wave 8 when information on waist circumference was not collected.

#### Complete Tooth Loss

The exposure was complete tooth loss collected in wave 0. Participants were asked: “Can I just check, do you still have some of your own teeth, or have you lost them all?” Based on their responses, participants were classified as dentate (those who reported having some natural teeth, coded as 0) or edentulous (those who reported having no natural teeth, coded as 1).

#### Confounders

We used data from wave 2 and treated confounders as time invariant. Education was determined by the highest qualification obtained. In the United Kingdom, the three-way education division is qualified to a level lower than “O-level” or equivalent (typically 0–11 years of schooling = low), qualified to a level lower than “A-level” or equivalent (typically 12–13 years of schooling = middle), and a higher qualification (typically >13 years of schooling = high). Wealth quintiles were calculated using nonpension wealth indicating financial, physical, and housing wealth net of debt (27). Smoking status was defined as nonsmoker, former smoker, or a current smoker. Frequency of alcohol consumption was classified as nondrinkers or drinking on 1 day a week, drinking on 2 to 6 days a week (frequently), or drinking daily. Self-reported physical activity was collected using three questions on the frequency of participation in vigorous, moderate, and mild-intensity physical activities. Self-reported physical activity was further categorized into the following two groups: sedentary life-style (no activity on a weekly basis) or active (mild, moderate, or vigorous activity at least once a week). We also included fruit and vegetable consumption as a time-varying indicator to test the mediating effect of diet on the association between complete tooth loss and AL, and participants were classified as consuming <5 and ≥5 portions a day. As previously described (28,29), fruit and vegetable consumption was initially assessed during the period 2006–2007 (wave 3) and then approximately every 2 years through 2012–2013 (wave 6). During waves 3 and 4, a series of questions assessed each respondent’s fruit intake during the previous day, with questions regarding a) how much various sized fruits were consumed (handfuls of very small, small, medium, large, and slices of very large fruit), b) how many tablespoons of various kinds of fruits were consumed (frozen or tinned, dried, or other mainly fruit dishes), and c) and how many small glasses of fruit juice were consumed. A similar series of questions were used to assess the previous day’s vegetable intake; respondents were asked how much salad was consumed (using a cereal bowl as the standard) and how many tablespoons of either vegetables or pulses (legumes) were consumed. The questionnaire changed during waves 5 and 6, and respondents were asked single questions about their fruit and vegetable intake. For fruit consumption, respondents were asked, “How many portions of fruit—of any kind—do you eat on a typical day? A portion of fruit is an apple or banana, a small bowl of grapes, or three tablespoons of tinned or stewed fruit. If you drink fruit juice, you can count one glass per day, but additional glasses of fruit juice do not count as additional portions.” For vegetable consumption, respondents were asked, “How many portions of vegetables—excluding potatoes—do you eat on a typical day? A serving or portion of vegetables means three heaped tablespoons of green or root vegetables such as carrots, parsnips, spinach, small vegetables like peas, baked beans or sweet corn, or a medium bowl of salad (lettuce, tomatoes, etc.).” To make fruit and vegetable consumption more consistent across all waves, answers from waves 3 and 4 were recoded to create summative variables that represented total daily intake. The number of portions of fruits and vegetables reported during each wave was added together to create one overall variable that represented the total number of fruit and vegetable portions eaten during a day.

### Statistical Analysis

All analyses were conducted in Stata version 15 (StataCorp LP, College Station, Texas). We first compared the baseline characteristics of dentate and edentulous participants using the χ^2^ test. We then compared AL scores at every wave between groups defined according to complete tooth loss and every confounder. Student’s *t* test was used to compare the AL score by complete tooth loss, sex, and fruit and vegetable consumption. For confounders with more than two categories (age groups, education, wealth, physical activity, smoking status, and alcohol consumption), linear trends in the AL score were tested by fitting each confounder as a continuous variable in linear regression models.

A linear mixed-effects (LME) model was fitted to model the 12-year change in AL score. LME models use all available outcome data over the follow-up period, handle unevenly spaced observations over time, and account for the fact that repeated measures on the same individual are correlated (30,31). We used wave information (coded as 0, 4, 8, and 12 for waves 2, 4, 6, and 8, respectively) as a continuous time indicator, with both its intercept and slope fitted as random effects to model individual variation in baseline value and rate of change in AL score, respectively. We did not impose any constraints during the estimation of the covariance matrix (i.e., unstructured). We first fitted a model without any confounders (null model) to determine the rate of change in AL score during the 12-year period of observation. We then tested the association of complete tooth loss with the 12-year change in AL score in three sequential models. The crude associations of complete tooth loss and each time-invariant confounder (sex, continuous age, education, wealth, physical activity, smoking status, and alcohol consumption) with the AL score were reported as model 1. The association of complete tooth loss with the AL score adjusted for all time-invariant confounders was reported as model 2. This model included the main effects of all predictors and the significant statistical interaction (product-term) of any predictor with the time indicator (wave). In this model, the estimates for each predictor represent its effect on the AL score at baseline (wave 2), whereas a significant interaction of that predictor with time represents its effect on the change in AL score over the 12-year period of observation (31). We used the likelihood ratio test to check whether the addition of any interaction term (one at a time) improved the goodness-of-fit of the model. The null hypothesis for the likelihood ratio test assumes that the interaction term is equal to zero and that the model containing only the main effects of predictors should be preferred (30). The role of diet in explaining the association between complete tooth loss and AL score was tested by comparing the estimate for complete tooth loss from model 2 with that from a model also containing the time-varying consumption of fruits and vegetables (model 3). We also tested the interaction of diet with time as described previously. We ran some sensitivity analyses to check the impact of some of our methodological decisions on the study findings. First, we included participants with complete AL data only to evaluate the impact of our imputation technique. Second, we recalculated AL scores using eight biomarkers only to evaluate the impact of rescaling AL score in wave 8 (when waist circumference was not collected). Third, we shortened the follow-up period to 8 years (waves 2, 4, and 6) to evaluate the impact of including AL data in wave 8 when only around half of cohort members were invited to the health examination.

Finally, to check whether specific AL biomarkers were driving the association, logistic mixed-effects models were fitted to evaluate the association of complete tooth loss with each separate dichotomized biomarker. Odds ratios (ORs) were thus reported. Two models were presented for each biomarker. Model 1 included the main effects of complete tooth loss adjusted for baseline confounders (demographic, socioeconomic, and behavioral factors). Model 2 also included the interaction between complete tooth loss and the time indicator (wave).

## RESULTS

We analyzed the data of 2430 adults (55% women), with a mean (standard deviation [SD]) age of 61.8 (8.3) years (range, 50–90 years). Participants excluded because of missing data were older, less educated, poorer, less physically active, and edentulous. The mean (SD) AL score was 3.5 (1.9; range, 0–9), 4.2 (2.1; range, 0–9), 4.0 (2.2; range, 0–9), and 4.0 (2.3; range, 0–9) in waves 2, 4, 6, and 8, respectively. In addition, 10.6% of participants were edentulous. Edentulous participants were more likely to be female, older, less educated, and poorer as well as more like to report current smoking, less physical activity, and lower consumption of alcohol and fruits and vegetables than dentate participants (Table 1).

**TABLE 1:** Baseline Characteristics of Participants With Different Levels of Complete Tooth Loss, the English Longitudinal Study of Ageing, 1998/1999/2001

	Study Sample (*n* = 2430), *n* (%)	Dentate (*n* = 2172), *n* (%)	Edentulous (*n* = 258), *n* (%)	*p^a^*
Sex				.007
Men	1087 (44.7)	992 (45.7)	95 (36.8)	
Women	1343 (55.3)	1180 (54.3)	163 (63.2)	
Age*^b^*, y				<.001
50–54	546 (22.5)	532 (24.5)	14 (5.4)	
55–64	1024 (42.1)	949 (43.7)	75 (29.1)	
65–74	644 (26.5)	531 (24.4)	113 (43.8)	
75+	216 (8.9)	160 (7.4)	56 (21.7)	
Education				<.001
None	1038 (42.7)	867 (39.9)	171 (66.3)	
Basic	658 (27.1)	606 (27.9)	52 (20.2)	
Higher	734 (30.2)	699 (32.2)	35 (13.6)	
Wealth				<.001
Q1 (poorest)	307 (12.6)	230 (10.6)	77 (29.8)	
Q2	411 (16.9)	337 (15.5)	74 (28.7)	
Q3	475 (19.5)	436 (20.1)	39 (15.1)	
Q4	627 (25.8)	587 (27.0)	40 (15.5)	
Q5 (wealthiest)	610 (25.1)	582 (26.8)	28 (10.9)	
Physical activity				<.001
Sedentary	29 (1.2)	19 (0.9)	10 (3.9)	
Low	506 (20.8)	411 (18.9)	95 (36.8)	
Moderate	1321 (54.4)	1199 (55.2)	122 (47.3)	
High	574 (23.6)	543 (25.0)	31 (12.0)	
Smoking status				<.001
Never	975 (40.1)	897 (41.3)	78 (30.2)	
Former	1139 (46.9)	1013 (46.6)	126 (48.8)	
Current	316 (13.0)	262 (12.1)	54 (20.9)	
Alcohol consumption				<.001
Never	192 (7.9)	151 (7.0)	41 (15.9)	
Rarely	656 (27.0)	551 (25.4)	105 (40.7)	
Frequently	1115 (45.9)	1039 (47.8)	76 (29.5)	
Daily	467 (19.2)	431 (19.8)	36 (14.0)	
Fruit and vegetable consumption				.039
<5 portions/d	1020 (42.0)	896 (41.3)	124 (48.1)	
5+ portions/d	1138 (46.8)	1031 (47.5)	107 (41.5)	

*^a^* χ^2^ Test was used for comparisons.

*^b^* Categories are for presentations purposes only.

Table 2 shows the crude associations of complete tooth loss and confounders with the AL score at every wave. In every wave, significant direct linear trends in the AL score were found according to age groups and smoking status, whereas significant inverse linear trends in the AL score were found according to education, wealth, physical activity, and alcohol consumption. Edentulous participants also had higher AL scores than did dentate participants in every wave. Men had lower AL scores than did women in waves 2 and 4, whereas participants eating 5+ portions a day of fruits and vegetables had lower AL scores than did those eating <5 portions a day in waves 4 and 6.

**TABLE 2:** Allostatic Load Scores by Baseline Complete Tooth Loss Status and Confounders at Every Wave of the English Longitudinal Study of Ageing

	Wave 2, Mean (SD)	Wave 4, Mean (SD)	Wave 6, Mean (SD)	Wave 8, Mean (SD)
Complete tooth loss				
Dentate	3.43 (1.86)	4.04 (2.11)	3.90 (2.19)	3.95 (2.28)
Edentulous	4.32 (1.88)	5.10 (2.02)	4.87 (2.03)	4.84 (2.35)
*p* Value for trend*^a^*	<.001	<.001	<.001	.004
Sex				
Men	3.48 (1.84)	4.13 (2.05)	3.93 (2.25)	3.99 (2.34)
Women	3.56 (1.92)	4.17 (2.18)	4.03 (2.15)	4.02 (2.26)
*p^b^*	.022	.048	.083	.738
Age groups*^c^*, y				
50–54	3.14 (1.81)	3.70 (2.17)	3.51 (2.19)	3.70 (2.17)
55–64	3.42 (1.93)	4.05 (2.13)	3.93 (2.17)	4.01 (2.33)
65–74	3.92 (1.87)	4.57 (2.05)	4.51 (2.16)	4.34 (2.33)
75+	3.76 (1.68)	4.51 (1.94)	4.13 (2.12)	4.21 (2.20)
*p* Value for trend*^a^*	<.001	<.001	<.001	<.001
Education				
None	3.85 (1.87)	4.50 (2.12)	4.40 (2.13)	4.36 (2.26)
Basic	3.36 (1.91)	3.98 (2.12)	3.75 (2.23)	3.94 (2.33)
Higher	3.21 (1.82)	3.81 (2.07)	3.65 (2.16)	3.68 (2.26)
*p* Value for trend*^a^*	<.001	<.001	<.001	<.001
Household wealth				
Q1 (poorest)	4.27 (1.93)	4.78 (2.21)	4.68 (2.24)	5.04 (2.40)
Q2	3.85 (1.89)	4.55 (2.24)	4.43 (2.20)	4.47 (2.30)
Q3	3.53 (1.84)	4.23 (2.01)	4.06 (2.13)	3.88 (2.21)
Q4	3.46 (1.84)	4.04 (2.11)	3.83 (2.14)	3.81 (2.25)
Q5 (wealthiest)	2.99 (1.77)	3.63 (1.97)	3.47 (2.12)	3.67 (2.23)
*p* Value for trend*^a^*	<.001	<.001	<.001	<.001
Physical activity				
Sedentary	4.09 (2.03)	5.23 (2.18)	4.95 (2.14)	5.40 (3.22)
Low	4.30 (1.99)	4.94 (2.23)	4.69 (2.23)	4.70 (2.22)
Moderate	3.44 (1.82)	4.06 (2.06)	3.97 (2.17)	3.99 (2.22)
High	3.01 (1.70)	3.60 (1.94)	3.44 (2.06)	3.61 (2.38)
*p* Value for trend*^a^*	<.001	<.001	<.001	<.001
Smoking status				
Never	3.34 (1.81)	3.98 (2.12)	3.81 (2.18)	3.78 (2.21)
Former	3.56 (1.91)	4.22 (2.10)	4.03 (2.19)	4.14 (2.34)
Current	3.98 (1.95)	4.47 (2.19)	4.35 (2.19)	4.30 (2.35)
*p* Value for trend*^a^*	<.001	<.001	<.001	.001
Alcohol drinking				
Never	4.10 (1.95)	4.79 (2.24)	4.47 (2.09)	4.71 (2.38)
Rarely	3.86 (1.91)	4.41 (2.17)	4.29 (2.17)	4.28 (2.31)
Frequently	3.33 (1.83)	3.99 (2.10)	3.77 (2.21)	3.86 (2.28)
Daily	3.32 (1.86)	3.92 (2.00)	3.90 (2.17)	3.82 (2.22)
*p* Value for trend*^a^*	<.001	<.001	<.001	<.001
Fruit and vegetable consumption*^c^*				
<5 portions/d	3.54 (1.90)	4.23 (2.10)	4.11 (2.21)	4.03 (2.27)
5+ portions/d	3.49 (1.90)	4.07 (2.13)	3.87 (2.17)	3.96 (2.28)
*p^b^*	.320	.005	.003	.204

*^a^* χ^2^ Test for linear trends was used.

*^b^* Student *t* test was used for comparison.

*^c^* Time-variant confounder. Cross-sectional associations at every wave are reported.

The null LME model showed an increase of 0.06 (95% CI = 0.05–0.06) units in the AL score per additional year of follow-up (Table 3). A small positive covariance was found between intercept and slope (0.03; 95% CI = 0.01–0.05), indicating that the largest increase in AL score was found among those with the highest baseline AL score. Complete tooth loss was positively associated with the AL score at baseline, with edentulous participants having 0.98 units (95% CI = 0.75–1.22 units) higher baseline AL score than dentate participants. This association was attenuated but remained significant after adjustments for confounders in model 2 (coefficient = 0.38; 95% CI = 0.14–0.62). Neither complete tooth loss (*p* = .557) nor any confounder (all, (*p* > .05) was significantly associated with the rate of change in AL score when added to the main effects model (Table S2, Supplemental Digital Content, http://links.lww.com/PSYMED/A724). Therefore, the final LME model only contained the main effects for all predictors. Predicted mean AL scores were calculated from the final LME model to show differences between dentate and edentulous participants over time. At the beginning of the follow-up (wave 2), the predicted mean AL scores were 3.60 (95% CI = 3.53–3.68) for dentate participants and 3.98 (95% CI = 3.76–4.21) for edentulous participants. At the end of the study (wave 8), the predicted mean AL scores were 4.28 (95% CI = 4.18–4.39) and 4.66 (95% CI = 4.42–4.90) for dentate and edentulous participants. The association of complete tooth loss with AL score remained unchanged after subsequent adjustment for the time-varying indicator of fruit and vegetable consumption in model 3 (coefficient = 0.40; 95% CI = 0.15–0.64). The interaction of fruit and vegetable consumption with time was not significant either (*p* > .05). Results from the different set of sensitivity analyses are shown in Table S3, Supplemental Digital Content, http://links.lww.com/PSYMED/A724. Similar findings were obtained when using participants with complete data on AL biomarkers, when using only eight biomarkers to estimate AL, and when excluding AL data from wave 8 to estimate changes over time.

**TABLE 3:** Linear Mixed-Effect Models for the Association Between Complete Tooth Loss and 12-Year Changes in Allostatic Load (*n* = 2430), the English Longitudinal Study of Ageing

	Model 1*^a^*, Coef. (95% CI)	Model 2*^a^*, Coef. (95% CI)	Model 3*^a^*, Coef. (95% CI)
Time*^b^*	0.06 (0.05 to 0.06)***	0.06 (0.05 to 0.06)***	0.06 (0.05 to 0.07)***
Complete tooth loss (reference: dentate)
Edentulous	0.98 (0.75 to 1.22)***	0.38 (0.14 to 0.62)**	0.40 (0.15 to 0.64)**
Sex (reference: men)
Women	0.05 (−0.09 to 0.20)	−0.12 (−0.26 to 0.03)	−0.15 (−0.30 to 0.00)
Age	0.03 (0.03 to 0.04)***	0.02 (0.01 to 0.03)***	0.02 (0.01 to 0.03)***
Education (reference: none)
Basic	−0.52 (−0.70 to −0.34)***	−0.19 (−0.37 to −0.01)*	−0.21 (−0.39 to −0.03)*
Higher	−0.69 (−0.86 to −0.52)***	−0.23 (−0.41 to −0.05)*	−0.27 (−0.45 to −0.08)**
Household wealth (reference: Q1, poorest)
Q2	−0.35 (−0.61 to −0.08)*	−0.18 (−0.44 to 0.08)	−0.23 (−0.50 to 0.03)
Q3	−0.66 (−0.92 to −0.40)***	−0.35 (−0.60 to −0.09)**	−0.38 (−0.64 to −0.12)**
Q4	−0.83 (−1.07 to −0.58)***	−0.40 (−0.65 to −0.16)**	−0.44 (−0.69 to −0.18)**
Q5 (wealthiest)	−1.22 (−1.46 to −0.97)***	−0.68 (−0.94 to −0.42)***	−0.73 (−0.99 to −0.46)***
Physical activity (reference: sedentary)
Low	−0.06 (−0.73 to 0.61)	0.20 (−0.45 to 0.85)	0.40 (−0.28 to 1.08)
Moderate	−0.92 (−1.58 to −0.26)**	−0.41 (−1.05 to 0.23)	−0.19 (−0.86 to 0.49)
High	−1.39 (−2.06 to −0.72)***	−0.78 (−1.43 to −0.12)*	−0.57 (−1.26 to 0.11)
Smoking status (reference: never)
Former	0.26 (0.10 to 0.42)**	0.23 (0.08 to 0.38)**	0.25 (0.09 to 0.40)**
Current	0.61 (0.38 to 0.84) ***	0.38 (0.15 to 0.61)**	0.43 (0.20 to 0.67) ***
Alcohol drinking (reference: never)
Rarely	−0.29 (−0.58 to 0.00)	−0.21 (−0.49 to 0.07)	−0.20 (−0.49 to 0.09)
Frequently	−0.78 (−1.06 to −0.50) ***	−0.42 (−0.70 to −0.15) **	−0.38 (−0.66 to −0.10)**
Daily	−0.78 (−1.08 to −0.47)***	−0.42 (−0.72 to −0.11) **	−0.36 (−0.67 to −0.05)*
Fruit and vegetable consumption (reference: <5 portions/d)				
5+ portions/d	0.01 (−0.07 to 0.09)		0.07 (−0.01 to 0.15)

Coef. (95% CI) = coefficient (95% confidence interval).

* *p* < .05.

** *p* < .01.

*** *p* < .001.

*^a^* Model 1 was unadjusted; model 2 was adjusted for demographic factors (sex and continuous age), SEP indicators (education and household wealth), and health behaviors (physical activity, smoking status, and alcohol drinking) at baseline (time-invariant); model 3 was additionally adjusted for diet as a potential mediator (consumption of fruits and vegetables treated as a time-varying confounder).

*^b^* Wave was used as the time indicator with four possible values (coded as 0, 4, 8, and 12 for waves 2, 4, 6, and 8). The coefficient indicates the change in allostatic load score per year increase in time.

Table 4 presents the models for the association of complete tooth loss with each separate AL biomarker. After adjustment for baseline confounders, complete tooth loss was associated with greater odds of having high systolic and diastolic blood pressures, fibrinogen, and waist circumference at baseline, after adjustment for confounders. The interaction between complete tooth loss and the time indicator (wave) was significant for three biomarkers, suggesting that complete tooth loss was positively associated with the rate of change in the probability of having high diastolic blood pressure, low high-density lipoprotein cholesterol, and high glycated hemoglobin over the 12-year period.

**TABLE 4:** Logistic Mixed-Effect Models for the Association of Complete Tooth Loss With Each Allostatic Load Biomarker (*n* = 2430), the English Longitudinal Study of Ageing

	Model 1		Model 2	
	OR	95% CI	OR	95% CI
Systolic blood pressure
Time*^a^*	0.90	0.78–1.04	0.90	0.78–1.04
Complete tooth loss (reference: dentate)
Edentulous	1.25	1.05–1.49**	1.23	1.00–1.51
Complete tooth loss by time			1.01	0.97–1.04
Diastolic blood pressure
Time	1.31	1.15–1.49**	1.31	1.15–1.49**
Complete tooth loss (reference: dentate)
Edentulous	1.16	1.01–1.32*	1.09	0.90–1.32
Complete tooth loss by time			1.06	1.03–1.09*
HDL cholesterol
Time	1.33	1.13–1.56**	1.34	1.14–1.56**
Complete tooth loss (reference: dentate)		
Edentulous	1.00	0.96–1.05	0.97	0.93–1.02
Complete tooth loss by time			1.02	1.01–1.03*
LDL cholesterol
Time	1.51	1.27–1.79**	1.51	1.27–1.79**
Complete tooth loss (reference: dentate)
Edentulous	0.97	0.93–1.02	0.98	0.93–1.03
Complete tooth loss by time			1.00	0.99–1.01
Triglycerides
Time	2.20	1.86–2.61***	2.21	1.87–2.62***
Complete tooth loss (reference: dentate)
Edentulous	1.14	0.71–1.82	1.00	0.95–1.06
Complete tooth loss by time			1.01	1.00–1.02
Fibrinogen
Time	1.26	1.08–1.47**	1.26	1.08–1.47**
Complete tooth loss (reference: dentate)
Edentulous	1.05	1.00–1.09*	1.05	1.00–1.10
Complete tooth loss by time			1.00	0.99–1.01
C-reactive protein
Time	1.70	1.45–2.00**	1.70	1.45–2.00**
Complete tooth loss (reference: dentate)
Edentulous	1.05	0.99–1.09	1.04	0.99–1.10
Complete tooth loss by time			1.00	0.99–1.02
Glycated hemoglobin
Time	1.15	1.03–1.27*	1.15	1.03–1.27*
Complete tooth loss (reference: dentate)
Edentulous	1.02	0.99–1.05	1.01	0.98–1.04
Complete tooth loss by time			1.01	1.00–1.01*
Waist circumference
Time	2.21	1.91–2.56***	2.21	1.91–2.56***
Complete tooth loss (reference: dentate)
Edentulous	1.04	1.00–1.09*	1.05	1.00–1.10*
Complete tooth loss by time			1.00	0.99–1.01

OR = odds ratios; CI = confidence interval; HDL = high-density lipoprotein; LDL = low-density lipoprotein.

* *p* < .05.

** *p* < .01.

*** *p* < .001.

*^a^* Wave was used as the time indicator with four possible values (coded as 0, 4, 8, and 12 for waves 2, 4, 6, and 8).

*^b^* Model 1 was adjusted for demographic factors (sex and continuous age), SEP indicators (education and household wealth), and health behaviors (physical activity, smoking status, and alcohol drinking) at baseline (time-invariant); model 2 was additionally adjusted for the interaction between complete tooth loss and the time indicator.

## DISCUSSION

This study shows that complete tooth loss was associated with greater AL scores at baseline but not with its rate of change over the 12-year-period, even after adjustment for demographic factors, socioeconomic position, and health behaviors. In addition, the consumption of fruits and vegetables did not play a role in explaining the association between complete tooth loss and AL scores.

The relationship between AL and oral health has been previously demonstrated (5). However, this association was based on periodontal disease with chronic systemic inflammation as a potential mediator for this association. This suggested mechanism refers to the inflammatory pathway whereby local inflammation (periodontal disease) may be linked to systemic inflammation (32). Inflammation, which is part of AL, plays an important role in the pathogenesis of atherosclerosis, and markers of low-grade inflammation have been consistently associated with a higher risk of cardiovascular disease. Not surprisingly, this pathway is less plausible in the current study sample because edentulous people do not have periodontal disease, although they may have had in the past. However, denture stomatitis may be a more likely source of oral inflammation among edentulous people (9). Analyses by biomarkers did not support the inflammatory pathway either because complete tooth loss was not associated with the rate of change in the probability of being in the risk category for fibrinogen or C-reactive protein. Rather, they pointed to dysregulation in the cardiovascular system (high diastolic blood pressure), lipid metabolism (low high-density lipoprotein cholesterol), and glucose metabolism (high glycated hemoglobin) as possible mechanisms driving the association with AL. These findings are more consistent with the nutritional pathway linking oral health with chronic conditions (20).

An alternative explanation is the measure of tooth loss used in this study. Complete tooth loss is a simple and irreversible measure of oral health. Ultimately, it represents total tooth mortality and reflects the accumulation of oral disease throughout the life course. It is therefore likely that most edentulous participants had been edentulous for several years before the baseline assessment. As such, complete tooth loss might not be the best early marker for accumulation of health deficits, whereas other measures of tooth loss, such as having a functional dentition or the number of teeth, may be more informative.

Complete tooth loss is associated with impaired masticatory function (19) and therefore poor nutritional status in older adults (7). Impaired nutritional status in terms of poor-quality diet has been linked with chronic conditions that are, in turn, risk factors for physical and cognitive decline later in life (16). However, our findings did not support a potential nutritional pathway because the consumption of fruits and vegetables did not explain the association between complete tooth loss and AL on one hand, and it was not associated with changes in AL scores on the other. It is possible that other dietary factors might play a stronger role in explaining changes in AL scores. There is evidence that diets rich in salt, meat, fat, and sugars are associated with higher AL scores (13), which might play a stronger role. Our analysis was somewhat limited by the availability of dietary data in ELSA.

The impact of adverse socioeconomic conditions and health risk behaviors (such as sugars intake, smoking, and access to health services) over the span of life are possible explanations for the observed association between complete tooth loss and AL at baseline. In other words, complete tooth loss and AL might share common determinants earlier in life. A person’s socioeconomic position at different stages of life course has been found to be associated with general health (33) and with an increased risk of complete tooth loss (34). Therefore, the life course approach has gained considerable attention in understanding social inequalities in oral conditions. Lifelong exposure to difficult circumstances translates itself into health consequences (25). This idea, originating in fundamental cause theory and the social determinants of health, underlies both the concept of AL (1) and the risk accumulation in life-course epidemiology (33). Risk accumulation is a way of characterizing exposures to health risks originating in the social location over the life course, resulting in accumulated chronic stress and material deprivation (25).

Our study has several strengths and potential limitations that need to be acknowledged. A major strength is the large nationally representative sample of community-dwelling English men and women 50 years or older using a wide range of confounders. To the best of our knowledge, this study is the first to investigate the association of complete tooth loss with AL changes. In addition, certified interviewers and qualified nurses following standardized protocols, thus assuring excellent quality of data, performed all measurements. The main limitation is that this study is observational rather than interventional, and as such, causality cannot be assumed. Regarding the oral health data, although self-report tooth loss is strongly correlated with clinical records (35), it is possible that more detailed measures, such as the number of teeth or having a functional dentition, might be more informative. Another potential source of limitation could be the use of self-reported fruit and vegetable consumption, which may not have been enough to capture the participants’ whole dietary intake. Without a full dietary assessment, information on calorie intake could not be estimated either. Therefore, the present findings await confirmation from further longitudinal studies addressing these issues.

## CONCLUSIONS

This study showed that complete tooth loss was associated with AL scores at baseline but not with changes in AL scores over time. In addition, the consumption of fruits and vegetables did not explain the association between complete tooth loss and AL scores. These findings highlight the role of common determinants of both conditions earlier in the life-span.

## Supplementary Material

SUPPLEMENTARY MATERIAL
