# Supplementary material for: Complete Tooth Loss and Allostatic Load Changes Later in Life: A 12-Year Follow-Up Analysis of the English Longitudinal Study of Ageing
Source: Psychosom Med. 2021 Apr 1;83(3):247–55. doi: 10.1097/PSY.0000000000000925 (PMC8016717; doi:10.1097/PSY.0000000000000925)
Supplement: SUPPLEMENTARY MATERIAL [file psymed-83-247-s002.docx]

**Complete tooth loss and allostatic load changes later in life: A 12-year follow-up analysis of the English Longitudinal Study of Ageing**

*Running head:* ***Complete tooth loss and allostatic load changes***

**Cesar de Oliveira, PhD - Correspoding author**

University College London, Department of Epidemiology & Public Health, English Longitudinal Study of Ageing (ELSA), 1-19 Torrington Place, WC1E 6BT, London-UK, Telephone: 07950 270289, Email: [c.oliveira@ucl.ac.uk](mailto:c.oliveira@ucl.ac.uk), ORCID: 0000-0002-4099-4762

**Wael Sabbah, PhD**

Division of Population & Patient Health, Dental Institute, King’s College London, London, UK.

**Ione Jayce Ceola Schneider, PhD**

Health Science Department and Rehabilitation Post-graduate Program, Federal University of Santa Catarina, Araranguá, Santa Catarina, Brazil.

**Eduardo Bernabé, PhD**

Division of Population & Patient Health, Dental Institute, King’s College London, London, UK.

**Number of Tables:** 4

**Supplemental Digital Content files:** 1

**Conflicts of Interest and Source of Funding**

The authors declare that they have no conflict of interest.

The English Longitudinal Study of Ageing was developed by a team of researchers based at the University College London, NatCen Social Research, the Institute for Fiscal Studies and the University of Manchester. The data were collected by NatCen Social Research. The funding is provided by National Institute on Aging Grant R01AG017644 and a consortium of UK government departments coordinated by the Economic and Social Research Council (ESRC). The funders had no involvement in the manuscript.

**Abstract**

**Objective:** There is little evidence of the association between complete tooth loss and allostatic load (AL). We investigated, firstly, the association between complete tooth loss and changes in AL over 12 years among older English adults. A second aim was to explore the role of fruit and vegetable consumption in explaining the above association. **Methods:** AL was calculated for 2,430 English Longitudinal Study of Ageing (ELSA) cohort (2004/5-2016/17) participants aged 50 years and older based on nine biomarkers: systolic and diastolic blood pressure, glycated haemoglobin, high- and low-density lipoprotein cholesterol, triglycerides, fibrinogen, C-reactive protein and waist circumference. The exposure was complete tooth loss. Participants were classified as dentate or edentulous. A linear mixed effects (LME) model was fitted to model the 12-year change in AL score and its association with complete tooth loss after adjustments for confounders (demographic factors, socioeconomic position and health behaviours). **Results:** Around 11% of the participants were edentulous. Complete tooth loss was positively associated with baseline AL scores but not with its rate of change over time. The predicted mean AL score was 3·60 (95%CI: 3·53, 3·68) and 3·98 (95%CI: 3·76, 4·21) as well as 4·28 (95%CI: 4·18, 4·39) and 4·66 (95%CI: 4·42, 4·90) for dentate and edentulous participants, at baseline and end of follow-up, respectively. Fruit and vegetable consumption was not associated with baseline AL or its rate of change. **Conclusions:** Complete tooth loss was associated with baseline AL score but not with its development over time whereas the consumption of fruit and vegetables did not help to explain this association. Both conditions may share common determinants earlier in life.

**Key words:** Tooth loss; allostatic load; biomarkers; metabolism; fruit and vegetable consumption; ageing

**List of abbreviations**

AL - Allostatic load

ELSA - English Longitudinal Study of Ageing

HSE - Health Survey for England

STROBE - Strengthening the Reporting of Observational Studies in Epidemiology

PA - Self-reported physical activity

LME - Linear mixed effects

LRT - Likelihood ratio test

SD - Standard deviation

95% CI - 95% confidence interval

**Introduction**

Allostatic load (AL) is the physiological wear and tear that the body sustains throughout the life course. Repeated exposures to chronic stressors trigger biological responses to cope with these stressors, leading to wear and tear on the immune, cardiovascular, metabolic and nervous systems and this is primarily marked by elevated epinephrine and cortisol levels (1). AL has been found to longitudinally predict self-rated health, physical function, frailty and, ultimately, mortality (2). It also shows gradients along markers of socioeconomic position (3). AL is, therefore, useful not only as a risk factor for morbidity, frailty and mortality, but also in understanding the mechanism of ageing throughout the life course (4). There is little evidence on the relationship between AL and oral health with most of the research arguing that AL predicts periodontal disease (5).

On the other hand, poor oral health, particularly complete tooth loss and periodontal diseases are considered an early marker of frailty (6), cognitive decline (7), metabolic syndrome (8-11) and mortality (12). The association between oral health and metabolic syndrome is particularly significant as the markers of AL include almost all markers of metabolic syndrome.

While earlier studies have depicted socioeconomic adversity and accumulation of health risk behaviours through lifespan as risk factors for the accumulation of biological markers of AL (13, 14), it is possible that oral health, particularly complete tooth loss, is a potential and often neglected risk factor or marker for the accumulation of AL. Complete tooth loss could impact some of the risk behaviours related to AL such as poor diet (15, 16). Diet is an important component of a healthy life as it has a role in the aetiology, and thus prevention, of many chronic conditions such as obesity, cardiovascular disease, diabetes and cancer among other chronic conditions (17, 18). Tooth loss reduces masticatory function and chewing ability, which in turn can limit food choices and variety in the diet (19). For these reasons, dietary intake has been regarded as an intermediate in the pathway between tooth retention and a number of diet-related chronic diseases (20). Complete tooth loss could also be an early marker of AL as it is linked to adverse socioeconomic conditions and risk behaviours (21, 22). One of the most consistent dietary correlates of tooth loss is a lower consumption of fruits and vegetables (23), which is also inversely associated with AL scores (13).

Given that complete loss of all teeth is still relatively common in Britain with 15% of adults aged 65 to 74, 30% of those aged 75 to 84 and 47% of those aged 85 and older edentulous (24), we set out to test whether complete tooth loss is associated with changes in allostatic load over a 12-year period among older English adults. A second aim was to explore the role of fruit and vegetable consumption in explaining the above association.

**Methods**

**Study population**

We used data from the English Longitudinal Study of Ageing (ELSA), a nationally representative panel study of adults aged 50+ living in private households in England. ELSA started in 2002-03 (wave 1) with a sample of 11,391 individuals drawn from households that had participated in the Health Survey for England (HSE) in 1998, 1999 or 2001 (known as ELSA wave 0). After wave 0, there have been 8 waves of data collection with follow-up interviews conducted biannually and health examinations conducted every 4 years.

For this study, we pooled together data on complete tooth loss collected in wave 0 with data on AL collected in waves 2 (2004-05), 4 (2008-09), 6 (2012-13) and 8 (2016-17). There were 2,511 participants with complete tooth loss data in wave 0 and allostatic load data for at least two of the above waves. Of them, 81 were excluded because of missing data on confounders (fruit and vegetable consumption=64, wealth=15, smoking=1, alcohol consumption=1). Therefore, the analytical sample included 2,430 participants (717 had allostatic load data on 4 waves, 793 on 3 waves and 920 on 2 waves).

All ELSA participants gave written informed consent. The National Research and Ethics Committee granted Ethical approval for all the ELSA waves (http://www.nres.npsa.nhs.uk/) (MREC/01/2/91). This study followed the Strengthening the Reporting of Observational Studies in Epidemiology (STROBE) reporting guideline.

**Variables**

**Allostatic load (AL)**

The outcome was AL, which was measured four times every 4 years. As a measure of multisystem dysregulation, AL should include biomarkers of the different systems that are thought to be affected by chronic stress exposure. AL was calculated based on 9 biomarkers: systolic and diastolic blood pressure (cardiovascular system); high- and low-density lipoprotein cholesterol and triglycerides (lipid metabolism); fibrinogen and C-reactive protein (inflammation); glycated haemoglobin (glucose metabolism) and waist circumference (body fat). Each biomarker was dichotomised according to clinical cut-off points (Table S1, Supplemental Digital Content, http://links.lww.com/PSYMED/A724) and a score for each participant was computed based on the number of biomarkers for which they were above the cut-off (risk category). This AL score ranged from 0 to 9, with higher values indicating higher multisystem physiological dysregulation. We accounted for current medication (2, 25). Participants with missing values in more than half (>5) of the 9 biomarkers were treated as missing when calculating the AL score. For those with <5 missing biomarkers, we averaged the available dichotomous indicators and multiplied this average by 9 to produce an equivalent scale range from 0 to 9 (26). The same approach was also used with all participants in wave 8 when information on waist circumference was not collected.

**Complete tooth loss**

The exposure was complete tooth loss collected in wave 0. Participants were asked: “Can I just check, do you still have some of your own teeth, or have you lost them all?” Based on their responses, participants were classified as dentate (those who reported having some natural teeth, coded as 0) or edentulous (those who reported having no natural teeth, coded as 1).

**Confounders**

We used data from wave 2 and treated confounders as time-invariant. Education was determined by the highest qualification obtained. In the United Kingdom, the 3-way education division is qualified to a level lower than “O-level” or equivalent (typically 0-11 years of schooling = low), qualified to a level lower than “A-level” or equivalent (typically 12-13 years of schooling = middle), and a higher qualification (typically >13 years of schooling = high). Wealth quintiles were calculated using non-pension wealth indicating financial, physical, and housing wealth net of debt (27). Smoking status was defined as non-smoker, former or a current smoker. Frequency of alcohol consumption was classified as: non-drinkers or drinking on one day a week, drinking on two to six days a week (frequently), or drinking daily. Self-reported physical activity (PA) was collected using three questions on the frequency of participation in vigorous, moderate and mild-intensity physical activities. PA was further categorized into the following two groups: sedentary lifestyle (no activity on a weekly basis) or active (mild, moderate or vigorous activity at least once a week). We also included fruit and vegetable consumption as a time-varying indicator to test the mediating effect of diet on the association between complete tooth loss and AL and participants were classified as consuming <5 and ≥5 portions a day. As previously described (28, 29), fruit and vegetables consumption was initially assessed during 2006-07 (wave 3) and then approximately every two years through 2012-13 (wave 6). During Waves 3 and 4, a series of questions assessed each respondent’s fruit intake during the previous day, with questions regarding (a) how much various sized fruits were consumed (handfuls of very small, small, medium, large, and slices of very large fruit), (b) how many tablespoons of various kinds of fruits were consumed (frozen or tinned, dried, or other mainly fruit dishes), and (c) and how many small glasses of fruit juice were consumed. A similar series of questions were used to assess the previous day’s vegetable intake; respondents were asked how much salad was consumed (using a cereal bowl as the standard) and how many tablespoons of either vegetables or pulses (legumes) were consumed. The questionnaire changed during Waves 5 and 6, and respondents were asked single questions about their fruit and vegetable intake. For fruit consumption, respondents were asked, “How many portions of fruit - of any kind - do you eat on a typical day? A portion of fruit is an apple or banana, a small bowl of grapes, or three tablespoons of tinned or stewed fruit. If you drink fruit juice, you can count one glass per day, but additional glasses of fruit juice do not count as additional portions.” For vegetable consumption, respondents were asked, “How many portions of vegetables - excluding potatoes - do you eat on a typical day? A serving or portion of vegetables means three heaped tablespoons of green or root vegetables such as carrots, parsnips, spinach, small vegetables like peas, baked beans or sweet corn, or a medium bowl of salad (lettuce, tomatoes, etc.).” To make fruit and vegetable consumption more consistent across all waves, answers from Waves 3 and 4 were recoded to create summative variables that represented total daily intake. The number of portions of fruits and vegetables reported during each wave were added together to create one overall variable that represented the total number of fruit and vegetable portions eaten during a day.

**Statistical analysis**

All analyses were conducted in Stata version 15 (StataCorp LP, College Station, TX). We first compared the baseline characteristics of dentate and edentulous participants using the Chi-squared test. We then compared AL scores at every wave between groups defined according to complete tooth loss and every confounder. Student’s t-test was used to compare the AL score by complete tooth loss, sex, and fruit and vegetable consumption. For confounders with more than 2 categories (age groups, education, wealth, physical activity, smoking status and alcohol consumption), linear trends in the AL score were tested by fitting each confounder as a continuous variable in linear regression models.

A linear mixed effects (LME) model was fitted to model the 12-year change in AL score. LME models use all available outcome data over the follow-up period, handle unevenly spaced observations over time and account for the fact that repeated measures on the same individual are correlated (30, 31). We used wave information (coded as 0, 4, 8 and 12 for waves 2, 4, 6 and 8 respectively) as a continuous time indicator, with both its intercept and slope fitted as random effects to model individual variation in baseline value and rate of change in AL score, respectively. We did not impose any constraints during the estimation of the covariance matrix (i.e. unstructured). We first fitted a model without any confounders (null model) to determine the rate of change in AL score during the 12-year period of observation. We then tested the association of complete tooth loss with the 12-year change in AL score in three sequential models. The crude associations of complete tooth loss and each time-invariant confounder (sex, continuous age, education, wealth, physical activity, smoking status and alcohol consumption) with the AL score were reported as Model 1. The association of complete tooth loss with the AL score adjusted for all time-invariant confounders was reported as Model 2. This model included the main effects of all predictors and the significant statistical interaction (product-term) of any predictor with the time indicator (wave). In this model, the estimates for each predictor represent its effect on the AL score at baseline (wave 2) whereas a significant interaction of that predictor with time represent its effect on the change in AL score over the 12-year period of observation (31). We used the likelihood ratio test (LRT) to check whether the addition of any interaction term (one at a time) improved the goodness-of-fit of the model. The null hypothesis for the LRT assumes that the interaction term is equal to zero and that the model containing only the main effects of predictors should be preferred (30). The role of diet in explaining the association between complete tooth loss and AL score was tested by comparing the estimate for complete tooth loss from Model 2 with that from a model also containing the time-varying consumption of fruits and vegetables (Model 3). We also tested the interaction of diet with time as described above. We ran some sensitivity analyses to check the impact of some of our methodological decisions on the study findings. First, we included participants with complete AL data only to evaluate the impact of our imputation technique. Second, we re-calculated AL scores using 8 biomarkers only to evaluate the impact of rescaling AL score in wave 8 (when waist circumference was not collected). Third, we shortened the follow-up period to 8 years (waves 2, 4 and 6) to evaluate the impact of including AL data in wave 8 when only around half of cohort members were invited to the health examination.

Finally, to check whether specific AL biomarkers were driving the association, logistic mixed effects models were fitted to evaluate the association of complete tooth loss with each separate dichotomised biomarker. Odds ratios (OR) were thus reported. Two models were presented for each biomarker. Model 1 included the main effects of complete tooth loss adjusted for baseline confounders (demographic, socioeconomic and behavioural factors). Model 2 also included the interaction between complete tooth loss and the time indicator (wave).

**Results**

We analysed data of 2,430 adults (55% women), with a mean age of 61·8 years (SD: 8·3, range: 50-90). Participants excluded because of missing data were older, less educated, poorer, less physically active and edentulous. The mean AL score was 3.5 (SD: 1·9, range: 0-9), 4·2 (SD: 2·1, range: 0-9), 4·0 (SD: 2·2, range 0-9) and 4.0 (SD: 2·3, range: 0-9) in waves 2, 4, 6 and 8, respectively. In addition, 10·6% of participants were edentulous. Edentulous participants were more likely to be female, older, less educated and poorer as well as more like to report current smoking, less physical activity, lower consumption of alcohol and fruits and vegetables than dentate participants (Table 1).

Table 2 shows the crude associations of complete tooth loss and confounders with the AL score at every wave. In every wave, significant direct linear trends in the AL score were found according to age groups and smoking status whereas significant inverse linear trends in the AL score were found according to education, wealth, physical activity and alcohol consumption. Edentulous participants also had higher AL scores than dentate participants in every wave. Men had lower AL scores than women in waves 2 and 4 whereas participants eating 5+ portions a day of fruits and vegetables had lower AL scores than those eating <5 portions a day in waves 4 and 6.

The null LME model showed an increase of 0·06 (95%CI: 0·05, 0·06) units in the AL score per additional year of follow-up (Table 3). A small positive covariance was found between intercept and slope (0·03; 95%CI: 0·01, 0·05), indicating that the largest increase in AL score was found among those with the highest baseline AL score. Complete tooth loss was positively associated with the AL score at baseline, with edentulous participants having 0·98 units (95%CI: 0·75, 1·22) higher baseline AL score than dentate participants. This association was attenuated but remained significant after adjustments for confounders in Model 2 (coefficient: 0·38; 95%CI: 0·14, 0·62). Neither complete tooth loss (p=0·557) nor any confounder (all p>0·05) was significantly associated with the rate of change in AL score when added to the main effects model (Table S2, Supplemental Digital Content, http://links.lww.com/PSYMED/A724). Therefore, the final LME model only contained the main effects for all predictors. Predicted mean AL scores were calculated from the final LME model to show differences between dentate and edentulous participants over time. At the beginning of the follow-up (wave 2), the predicted mean AL score was 3·60 (95%CI: 3·53, 3·68) for dentate participants and 3·98 (95%CI: 3·76, 4·21) for edentulous participants. At the end of the study (wave 8), the predicted mean AL score was 4·28 (95%CI: 4·18, 4·39) and 4·66 (95%CI: 4·42, 4·90) for dentate and edentulous participants. The association of complete tooth loss with AL score remain unchanged after subsequent adjustment for the time-varying indicator of fruit and vegetable consumption in Model 3 (coefficient: 0 40; 95%CI: 0·15, 0·64). The interaction of fruit and vegetable consumption with time was not significant either (p>0·05). Results from the different set of sensitivity analyses are shown in Table S3, Supplemental Digital Content, http://links.lww.com/PSYMED/A724. Similar findings were obtained when using participants with complete data on AL biomarkers, when using only 8 biomarkers to estimate AL and when excluding AL data from wave 8 to estimate changes over time.

Table 4 presents the models for the association of complete tooth loss with each separate AL biomarker. After adjustment for baseline confounders, complete tooth loss was associated with greater odds of having high systolic and diastolic blood pressure, fibrinogen and waist circumference at baseline, after adjustment for confounders. The interaction between complete tooth loss and the time indicator (wave) was significant for three biomarkers, suggesting that complete tooth loss was positively associated with the rate of change in the probability of having high diastolic blood pressure, low HDL cholesterol and high glycated haemoglobin over the 12-year period.

**Discussion**

This study shows that complete tooth loss was associated with greater allostatic load scores at baseline but not with its rate of change over the 12-year-period, even after adjustment for demographic factors, socioeconomic position and health behaviours. In addition, the consumption of fruits and vegetables did not play a role in explaining the association between complete tooth loss and allostatic load scores.

The relationship between AL and oral health has been previously demonstrated (5). However, this association was based on periodontal disease with chronic systemic inflammation as a potential mediator for this association. This suggested mechanism refers to the inflammatory pathway whereby local inflammation (periodontal disease) may be linked to systemic inflammation (32). Inflammation which is part of AL plays an important role in the pathogenesis of atherosclerosis, and markers of low-grade inflammation have been consistently associated with a higher risk of cardiovascular disease. Not surprisingly, this pathway is less plausible in the current study sample because edentulous people do not have periodontal disease, although they may have had in the past. However, denture stomatitis may be a more likely source of oral inflammation among edentulous people (9). Analyses by biomarkers did not support the inflammatory pathway either as complete tooth loss was not associated with the rate of change in the probability of being in the risk category for fibrinogen or C-reactive protein. Rather, they pointed to dysregulation in the cardiovascular system (high diastolic blood pressure), lipid metabolism (low HDL cholesterol) and glucose metabolism (high glycated haemoglobin) as possible mechanisms driving the association with AL. These findings are more consistent with the nutritional pathway linking oral health with chronic conditions (20).

An alternative explanation is the measure of tooth loss used in this study. Complete tooth loss is a simple and irreversible measure of oral health. Ultimately, it represents total tooth mortality and reflects the accumulation of oral disease throughout the life course. It is therefore likely that most edentulous participants had been edentulous for several years before the baseline assessment. As such, complete tooth loss might not be the best early marker for accumulation of health deficits, whereas other measures of tooth loss, such as having a functional dentition or the number of teeth, may be more informative.

Complete tooth loss is associated with impaired masticatory function (19) and therefore poor nutritional status in older adults (7). Impaired nutritional status in terms of poor-quality diet has been linked with chronic conditions that are, in turn, risk factors for physical and cognitive decline later in life (16). However, our findings did not support a potential nutritional pathway because the consumption of fruits and vegetables did not explain the association between complete tooth loss and AL, on one hand, and it was not associated with changes in AL scores, on the other. It is possible that other dietary factors might play a stronger role in explaining changes in AL scores. There is evidence that diets rich in salt, meat, fat and sugars are associated with higher AL scores (13), which might play a stronger role. Our analysis was somewhat limited by the availability of dietary data in ELSA.

The impact of adverse socioeconomic conditions and health risk behaviours (such as sugars intake, smoking and access to health services) over the span of life are possible explanations for the observed association between complete tooth loss and allostatic load at baseline. In other words, complete tooth loss and allostatic load might share common determinants earlier in life. A person’s socioeconomic position at different stages of life course has been found to be associated with general health (33) and with an increased risk of complete tooth loss (34). Therefore, the life course approach has gained considerable attention in understanding social inequalities in oral conditions. Lifelong exposure to difficult circumstances translates itself into health consequences (25). This idea, originating in fundamental cause theory and the social determinants of health underlies both the concept of allostatic load (1) and the risk accumulation in life-course epidemiology (33). Risk accumulation is a way of characterising exposures to health risks originating in the social location over the life course, resulting in accumulated chronic stress and material deprivation (25).

Our study has several strengths and potential limitations that need to be acknowledged. A major strength is the large nationally representative sample of community-dwelling English men and women aged 50 years or older using a wide range of confounders. To the best of our knowledge, this study is the first to investigate the association of complete tooth loss with AL changes. In addition, certified interviewers and qualified nurses following standardised protocols, thus assuring excellent quality of data, performed all measurements. The main limitation is that this study is observational rather than interventional, and as such causality cannot be assumed. Regarding the oral health data, although self-report tooth loss is strongly correlated with clinical records (35), it is possible that more detailed measures, such as the number of teeth or having a functional dentition, might be more informative. Another potential source of limitation could be the use of self-reported fruit and vegetable consumption which may not have been enough to capture the participants’ whole dietary intake. Without a full dietary assessment, information on calorie intake could not be estimated either. Therefore, the present findings await confirmation from further longitudinal studies addressing these issues.

**Conclusions**

This study showed that complete tooth loss was associated with AL scores at baseline but not with changes in AL scores over time. In addition, the consumption of fruits and vegetables did not explain the association between complete tooth loss and AL scores. These findings highlight the role of common determinants of both conditions earlier in the life span.

**Ethics approval and consent to participate**

All ELSA participants gave written informed consent. The National Research and Ethics Committee granted Ethical approval for all the ELSA waves (http://www.nres.npsa.nhs.uk/) (MREC/01/2/91). This study followed the Strengthening the Reporting of Observational Studies in Epidemiology (STROBE) reporting guideline.

**Consent for publication**

Not applicable.

**Availability of data and materials**

The English Longitudinal Study of Ageing data are available to the scientific community from the UK Data Service for researchers who meet the criteria for access to confidential data, under conditions of the End User License http://ukdataservice.ac.uk/media/455131/cd137-enduserlicence.pdf. The data can be accessed from https://beta.ukdataservice.ac.uk/datacatalogue/series/series?id=200011#!/access-data. Contact with the UK Data Service regarding access to the English Longitudinal Study of Ageing can be made through the website https://www.ukdataservice.ac.uk/about-us/contact, by phone +44 (0)1206 872143 or by email at [help@ukdataservice.ac.uk](mailto:help@ukdataservice.ac.uk).

**Competing interests**

The authors declare that they have no conflict of interest.

**Funding**

The English Longitudinal Study of Ageing was developed by a team of researchers based at the University College London, NatCen Social Research, the Institute for Fiscal Studies and the University of Manchester. The data were collected by NatCen Social Research. The funding is provided by National Institute on Aging Grant R01AG017644 and a consortium of UK government departments coordinated by the Economic and Social Research Council (ESRC). The funders had no involvement in the manuscript.

**Authors' contributions**

CO and EB conceptualised and designed the study. EB carried out the analyses and takes responsibility for the integrity of the data and the accuracy of the data analysis. CO, EB, WS and IJCS contributed to the interpretation of the results, drafted and revised the Article. All authors approved the final Article as submitted and agree to be accountable for all aspects of the work.

**Acknowledgements**

The ELSA study was developed by researchers based at University Colleague London, the Institute of Fiscal Studies and the National Centre for Social Research.

**References**

1. McEwen BS. Stress, adaptation, and disease. Allostasis and allostatic load. Ann N Y Acad Sci. 1998;840:33-44.

2. Read S, Grundy E. Allostatic load and health in the older population of England: a crossed-lagged analysis. Psychosomatic medicine. 2014;76:490-6.

3. Tampubolon G, Maharani A. Trajectories of allostatic load among older Americans and Britons: longitudinal cohort studies. BMC Geriatr. 2018;18:255.

4. Gruenewald TL, Karlamangla AS, Hu P, Stein-Merkin S, Crandall C, Koretz B, Seeman TE. History of socioeconomic disadvantage and allostatic load in later life. Soc Sci Med. 2012;74:75-83.

5. Sabbah W, Gomaa N, Gireesh A. Stress, allostatic load, and periodontal diseases. Periodontol 2000. 2018;78:154-61.

6. Hakeem FF, Bernabé E, Sabbah W. Association between oral health and frailty: A systematic review of longitudinal studies. Gerodontology. 2019;36:205-15.

7. Tsakos G, Watt RG, Rouxel PL, de Oliveira C, Demakakos P. Tooth loss associated with physical and cognitive decline in older adults. J Am Geriatr Soc. 2015;63:91-9.

8. Kim SW, Cho KH, Han KD, Roh YK, Song IS, Kim YH. Tooth Loss and Metabolic Syndrome in South Korea: The 2012 Korean National Health and Nutrition Examination Survey. Medicine (Baltimore). 2016;95:e3331.

9. Saito M, Shimazaki Y, Nonoyama T, Tadokoro Y. Number of Teeth, Oral Self-care, Eating Speed, and Metabolic Syndrome in an Aged Japanese Population. J Epidemiol. 2019;29:26-32.

10. Shin HS. The Number of Teeth Is Inversely Associated With Metabolic Syndrome: A Korean Nationwide Population-Based Study. J Periodontol. 2017;88:830-8.

11. Zhu Y, Hollis JH. Associations between the number of natural teeth and metabolic syndrome in adults. J Clin Periodontol. 2015;42:113-20.

12. Romandini M, Baima G, Antonoglou G, Bueno J, Figuero E, Sanz M. Periodontitis, Edentulism, and Risk of Mortality: A Systematic Review with Meta-analyses. J Dent Res. 2020:22034520952401.

13. Suvarna B, Suvarna A, Phillips R, Juster RP, McDermott B, Sarnyai Z. Health risk behaviours and allostatic load: A systematic review. Neurosci Biobehav Rev. 2020;108:694-711.

14. Vineis P, Delpierre C, Castagné R, Fiorito G, McCrory C, Kivimaki M, Stringhini S, Carmeli C, Kelly-Irving M. Health inequalities: Embodied evidence across biological layers. Soc Sci Med. 2020;246:112781.

15. Gaewkhiew P, Sabbah W, Bernabé E. Does tooth loss affect dietary intake and nutritional status? A systematic review of longitudinal studies. J Dent. 2017;67:1-8.

16. Kiesswetter E, Poggiogalle E, Migliaccio S, Donini LM, Sulmont-Rossé C, Feart C, Suwalska A, Wieczorowska-Tobis K, Pałys W, Łojko D, Saba A, Sinesio F, Polito A, Moneta E, Ciarapica D, Brug J, Volkert D. Functional determinants of dietary intake in community-dwelling older adults: a DEDIPAC (DEterminants of DIet and Physical ACtivity) systematic literature review. Public Health Nutr. 2018;21:1886-903.

17. Estruch R, Ros E. The role of the Mediterranean diet on weight loss and obesity-related diseases. Rev Endocr Metab Disord. 2020;21:315-27.

18. Cena H, Calder PC. Defining a Healthy Diet: Evidence for The Role of Contemporary Dietary Patterns in Health and Disease. Nutrients. 2020;12.

19. Tada A, Miura H. Systematic review of the association of mastication with food and nutrient intake in the independent elderly. Arch Gerontol Geriatr. 2014;59:497-505.

20. Ritchie CS, Joshipura K, Hung HC, Douglass CW. Nutrition as a mediator in the relation between oral and systemic disease: associations between specific measures of adult oral health and nutrition outcomes. Crit Rev Oral Biol Med. 2002;13:291-300.

21. Hach M, Christensen LB, Lange T, Hvidtfeldt UA, Danielsen B, Diderichsen F, Osler M, Prescott E, Andersen I. Social inequality in tooth loss, the mediating role of smoking and alcohol consumption. Community Dent Oral Epidemiol. 2019;47:416-23.

22. Starr JM, Pattie A, Whalley LJ, Deary IJ. Predictors of tooth loss in the 1921 Lothian Birth Cohort. Age Ageing. 2008;37:111-4.

23. Kossioni AE. The Association of Poor Oral Health Parameters with Malnutrition in Older Adults: A Review Considering the Potential Implications for Cognitive Impairment. Nutrients. 2018;10.

24. Steele JG, Treasure ET, O'Sullivan I, Morris J, Murray JJ. Adult Dental Health Survey 2009: transformations in British oral health 1968-2009. Br Dent J. 2012;213:523-7.

25. van Deurzen I, Vanhoutte B. A Longitudinal Study of Allostatic Load in Later Life: The Role of Sex, Birth Cohorts, and Risk Accumulation. Research on aging. 2019;41:419-42.

26. Carroll JE, Gruenewald TL, Taylor SE, Janicki-Deverts D, Matthews KA, Seeman TE. Childhood abuse, parental warmth, and adult multisystem biological risk in the Coronary Artery Risk Development in Young Adults study. Proceedings of the National Academy of Sciences of the United States of America. 2013;110:17149-53.

27. Demakakos P, Biddulph JP, Bobak M, Marmot MG. Wealth and mortality at older ages: a prospective cohort study. J Epidemiol Community Health. 2016;70:346-53.

28. Boehm JK, Soo J, Zevon ES, Chen Y, Kim ES, Kubzansky LD. Longitudinal associations between psychological well-being and the consumption of fruits and vegetables. Health psychology : official journal of the Division of Health Psychology, American Psychological Association. 2018;37:959-67.

29. Kobayashi LC, Steptoe A. Social Isolation, Loneliness, and Health Behaviors at Older Ages: Longitudinal Cohort Study. Ann Behav Med. 2018;52:582-93.

30. Singer JD, Willett JB. Applied Longitudinal Data Analysis: Modeling Change and Event Occurrence. Oxford: Oxford University Press; 2003.

31. Twisk JWR. Applied Longitudinal Data Analysis for Epidemiology: A Practical Guide. New York, NY: Cambridge University Press; 2013.

32. de Oliveira C, Watt R, Hamer M. Toothbrushing, inflammation, and risk of cardiovascular disease: results from Scottish Health Survey. Bmj. 2010;340:c2451.

33. Ben-Shlomo Y, Cooper R, Kuh D. The last two decades of life course epidemiology, and its relevance for research on ageing. International journal of epidemiology. 2016;45:973-88.

34. Ramsay SE, Papachristou E, Watt RG, Lennon LT, Papacosta AO, Whincup PH, Wannamethee SG. Socioeconomic disadvantage across the life-course and oral health in older age: findings from a longitudinal study of older British men. J Public Health (Oxf). 2018;40:e423-e30.

35. Andrade FB, Antunes JLF, Souza Junior PRB, Lima-Costa MF, Oliveira C. Life course socioeconomic inequalities and oral health status in later life: ELSI-Brazil. Rev Saude Publica. 2018;52Suppl 2:7s.
