# Supplementary material for: Complete Tooth Loss and Allostatic Load Changes Later in Life: A 12-Year Follow-Up Analysis of the English Longitudinal Study of Ageing
Source: Psychosom Med. 2021 Apr 1;83(3):247–55. doi: 10.1097/PSY.0000000000000925 (PMC8016717; doi:10.1097/PSY.0000000000000925)
Supplement: SUPPLEMENTARY MATERIAL [file psymed-83-247-s003.docx]

**Table S1.** Cut-off point for the risk category in each biomarker used to construct the allostatic load (AL) score.

| **Biomarkers** | | **Cut-off point** |
| --- | --- | --- |
| *Cardiovascular system* | |  |
|  | Systolic blood pressure | High: >=140 mm/Hg |
|  | Diastolic blood pressure | High: >=90 mm/Hg |
| *Lipid metabolism* | |  |
|  | HDL cholesterol | Low: <40 mg/dl male; <50 mg/dl female |
|  | LDL cholesterol | High: >=100 mg/dl |
|  | Triglycerides | High: >=150 mg/dl |
| *Inflammation* | |  |
|  | Fibrinogen | High risk: >3.7g/l |
|  | C-reactive protein (CRP) | High risk: >3 mg/l |
| *Glucose metabolism* | |  |
|  | glycated haemoglobin (HbA1c) | High: >=6.5% |
| *Body fat* | |  |
|  | Waist circumference | High: >102 male; >88 female |

**Table S2.** Likelihood ratio tests for the interaction of each predictor with the time indicator (wave) when added to the main effects model

| **Models tested**^a^ | **Log-likelihood** | **Likelihood ratio test** | | |
| --- | --- | --- | --- | --- |
|  |  | **df** | **Value** | **p value** |
| M2 (main effects only, reference model) | -13858.2 | 23 |  |  |
| M2 + Complete tooth loss X Time | -13858.0 | 24 | 0.35 | 0.557 |
| M2 + Education X Time | -13856.7 | 25 | 2.90 | 0.234 |
| M2 + Household wealth X time | -13855.5 | 27 | 5.46 | 0.243 |
| M2 + Smoking status X Time | -13857.3 | 25 | 1.71 | 0.424 |
| M2 + Alcohol drinking X Time | -13856.8 | 26 | 2.89 | 0.409 |
| M2 + Physical activity X Time | -13855.9 | 26 | 4.57 | 0.206 |
| M2 + Sex X Time | -13856.6 | 24 | 3.10 | 0.078 |
| M2 + Age X Time | -13857.9 | 24 | 0.52 | 0.473 |

^a^ Model 2 (M2) included demographic factors (sex and continuous age), SEP indicators (education and household wealth) and health behaviours (physical activity, smoking status and alcohol drinking) and complete tooth loss at baseline (time-invariant) as predictors.

**Table S3.** Results from sensitivity analyses for the association between baseline complete tooth loss, time-varying consumption of fruits and vegetables and 12-year change in AL score, The English Longitudinal Study of Ageing (ELSA).

|  | | | **Model 1^b^** |  | **Model 2^b^** |  | **Model 3^b^** |
| --- | --- | --- | --- | --- | --- | --- | --- |
|  |  |  | **Coef. (95% CI)** |  | **Coef. (95% CI)** |  | **Coef. (95% CI)** |
| **Sensitivity analysis 1: Complete data analysis (n=2040)** | | | | | |  |  |
|  | *Time (years)^a^* | | 0·05 (0·04, 0·06)‡ |  | 0·06 (0·05, 0·06)‡ |  | 0·06 (0·05, 0·07)‡ |
|  | *Complete tooth loss (reference: Dentate)* | | |  |  |  |  |
|  |  | Edentulous | 0·97 (0·70, 1·24)‡ |  | 0·44 (0·17, 0·71)† |  | 0·48 (0·20, 0·76)† |
|  | *Fruits and vegetables consumption (reference: <5 portions/day)* | | | | |  |  |
|  |  | 5+ portions/day | 0·01 (-0·10, 0·08) |  | ·· |  | 0·04 (-0·05, 0·13) |
| **Sensitivity analysis 2: Excluding wave 2 outcome data (n=2419)** | | | | | |  |  |
|  | *Time (years)* | | 0·06 (0·05, 0·06)‡ |  | 0·06 (0·05, 0·06)‡ |  | 0·06 (0·05, 0·07)‡ |
|  | *Complete tooth loss (reference: Dentate)* | | |  |  |  |  |
|  |  | Edentulous | 1·00 (0·76, 1·23)‡ |  | 0·40 (0·16, 0·64)† |  | 0·42 (0·17, 0·67)† |
|  | *Fruits and vegetables consumption (reference: <5 portions/day)* | | | | |  |  |
|  |  | 5+ portions/day | 0·01 (-0·08, 0·09) |  | ·· |  | 0·07 (-0·02, 0·16) |
| **Sensitivity analysis 3: Excluding wave 8 outcome data (n=2368)** | | | | | |  |  |
|  | *Time (years)* | | 0·07 (0·06, 0·08)‡ |  | 0·07 (0·06, 0·08)‡ |  | 0·07 (0·06, 0·08)‡ |
|  | *Complete tooth loss (reference: Dentate)* | | |  |  |  |  |
|  |  | Edentulous | 0·84 (0·63, 1·05)‡ |  | 0·29 (0·08, 0·50)† |  | 0·32 (0·10, 0·54)† |
|  | *Fruits and vegetables consumption (reference: <5 portions/day)* | | | | |  |  |
|  |  | 5+ portions/day | 0·01 (-0·06, 0·08) |  | ·· |  | 0·06 (-0·01, 0·14) |

^a^ Wave was used as the time indicator with four possible values (coded as 0, 4, 8 and 12 for waves 2, 4, 6 and 8). The coefficient indicates the change in AL score per year increase in time.

^b^ Model 1 was unadjusted; Model 2 was adjusted for demographic factors (sex and continuous age), SEP indicators (education and household wealth) and health behaviours (physical activity, smoking status and alcohol drinking) at baseline (time-invariant); Model 3 was additionally adjusted for diet as a potential mediator (consumption of fruits and vegetables treated as a time-varying covariate).

* p<0.05, † p<0.01, ‡ p<0.001
